# PFC Simulation and Experimental Investigation of Crack Evolution and Surface Morphology in Rehbinder-Effect-Assisted Longitudinal–Torsional Ultrasonic Vibration Milling of Borosilicate Glass

**DOI:** 10.3390/ma19143081

**Published:** 2026-07-17

**Authors:** Jie Yi, Rui Wang, Tao Wang, Xiaojie Liu, Cuicui Li, Junfeng Xiang

**Affiliations:** 1 School of Mechanical and Electronic Engineering, Shandong Jianzhu University, Jinan 250101, China; yijie18@sdjzu.edu.cn (J.Y.);; 2 Institute of Ultrasonic Technology, Institute of Intelligent Manufacturing Technology, Shenzhen Polytechnic University, Shenzhen 518055, China; 3 Shandong Zhangqiu Blower Co., Ltd., Jinan 250200, China; licuicui@blower.cn; 4 School of Civil Aviation, Northwestern Polytechnical University, Xi’an 710072, China

**Keywords:** borosilicate glass, discrete element method, PFC2D, longitudinal–torsional ultrasonic vibration-assisted milling, Rehbinder effect, surface roughness

## Abstract

Borosilicate glass is widely used in microfluidic chips, optical components, and precision devices, but it is susceptible to cracking and subsurface damage during machining because of its high hardness and brittleness. Longitudinal–torsional ultrasonic vibration-assisted milling (LTUVAM) can improve material removal stability, whereas the Rehbinder effect can reduce surface energy and weaken local material resistance. In this study, the additional effect of a Rehbinder-active medium under LTUVAM conditions was investigated to reduce crack-related surface damage and improve surface integrity. First, a two-dimensional cutting model of borosilicate glass was established using Particle Flow Code in two dimensions (PFC2D) to simulate crack initiation and propagation under conventional milling (CM) and LTUVAM conditions. The simulation results show that ultrasonic vibration promoted finer and more distributed microcracks and reduced the development of large-scale fractures, thereby modifying the brittle material removal behavior. Subsequently, milling experiments were conducted under LTUVAM conditions with a Rehbinder-active medium supplied to the cutting zone. Compared with LTUVAM without the solution, the proposed process significantly reduced surface roughness, with maximum reductions of 40.7% in *S_a_* and 32.3% in *S_q_* at a cutting depth of 50 μm. Finally, the simulated force variation and crack evolution were qualitatively compared with the experimental roughness trends and reported results, showing consistent mechanism-level trends. This work provides a theoretical and experimental basis for improving the machining quality of hard and brittle materials through the coupling of ultrasonic vibration and surface-active effects.

## 1. Introduction

Borosilicate glass is widely used in medical testing, optical components, industrial microfluidic chips and other fields due to its excellent light transmission performance, chemical stability and thermal stability. However, this material features high hardness and brittleness, and is prone to cracking, chipping and surface damage during conventional machining processes. As a result, its surface and subsurface quality are difficult to ensure, which seriously impairs the optical performance and service life of the components [1]. Therefore, conducting research on high-efficiency and precision machining methods for borosilicate glass holds important theoretical significance and engineering value.

**Figure 1:**
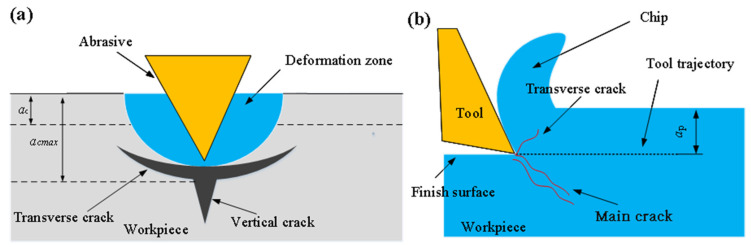
Crack evolution during machining. (**a**) crack types during machining; (**b**) schematic illustration of machining–induced cracks.

In recent years, with the continuous expansion of application fields for hard and brittle materials, the limitations of conventional processing methods such as cutting, grinding and chemical mechanical polishing (CMP) have become increasingly prominent in terms of machining accuracy and efficiency. To improve machining quality and mitigate stress-induced crack propagation during material removal, various advanced machining technologies have been proposed. Surface chipping and subsurface damage in borosilicate glass are mainly governed by crack initiation and propagation. During machining, increasing cutting depth and accumulated stress promote the initiation of vertical cracks perpendicular to the machined surface, which first form inside the material; as machining proceeds, transverse cracks parallel to the machining surface form within the material, as shown in Figure 1. Among them, the downward propagation of vertical cracks leads to subsurface damage, while the outward extension of transverse cracks intersects with the machining surface, resulting in surface breakage [2].

From the broader perspective of precision milling, Yi et al. [3,4,5,6,7] investigated force prediction, deformation control, residual-stress evolution and chatter behavior in micro-milling of thin-walled or curved metallic structures. Xiang et al. [8] further analyzed milling-induced surface morphology and residual stress under coupled milling and ultrasonic surface rolling. These studies demonstrate that cutting-force variation, dynamic tool–workpiece interaction and surface-integrity evolution are important issues in precision milling; however, because their target materials and deformation mechanisms differ from those of brittle glass, they are cited here only as a methodological reference for the subsequent investigation of borosilicate glass machining.

For borosilicate glass and related silicate glasses, material removal is highly sensitive to brittle fracture, crack-network evolution and the ductile–brittle transition; therefore, machining strategies should be discussed from both mechanical-damage and surface-chemical perspectives. In terms of glass machining processes, Jahan et al. [9] reviewed conventional, non-conventional and hybrid micromachining methods for glass materials, and summarized the applicability of cutting, grinding/lapping, polishing, chemical mechanical polishing (CMP), laser-based machining and hybrid processes in glass component fabrication. Their review indicates that glass machining is strongly constrained by edge chipping, surface pits, radial/median cracks, lateral cracks and subsurface damage, which makes material-specific damage suppression essential for maintaining optical and functional performance. From the perspective of surface chemistry, Liang et al. [10] investigated the effects of chemical additives on CMP of glass substrates and showed that chemically active media can influence glass removal behavior through surface reactions, slurry dispersion, enhanced wetting and polishing efficiency. Regarding crack-related damage mechanisms in glassy materials, Widerhorn and Fuller [11] analyzed the influence of surface forces on subcritical crack growth in glass, demonstrating that adsorption-related surface interactions can modify crack-growth kinetics. Ciccotti [12] further reviewed stress-corrosion mechanisms in silicate glasses and clarified the role of water-assisted chemical reactions at crack tips in weakening siloxane bonds and promoting subcritical crack propagation. Regarding adsorption-induced weakening, Malkin [13] summarized the regularities and mechanisms of the Rehbinder effect, indicating that adsorption-active environments can reduce surface or interfacial energy and facilitate deformation or fracture in solids. Flyagina et al. [14] used quantum-chemical simulation to reveal the adsorption-induced reduction in the strength of siloxane bonds, providing molecular-level evidence for the Rehbinder-type effect in silicate systems. These studies suggest that the surface integrity of machined borosilicate glass is governed by the coupled effects of crack evolution, ductile–brittle transition and surface chemical interactions. Nevertheless, the synergistic influence of ultrasonic vibration and adsorption-active media during borosilicate glass milling, especially its relationship with crack evolution and three-dimensional surface integrity, remains insufficiently clarified.

**Figure 2:**
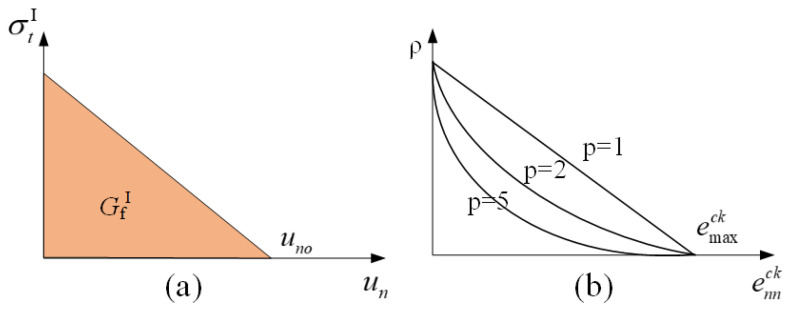
Finite element fracture model considering tensile and shear behaviors. (**a**) Fracture energy based cracking model for Mode I; (**b**) Shear retention model with power law form for Mode II.

Cracks are the key factor causing machining damage to hard and brittle materials; therefore, understanding crack initiation and propagation is essential for developing high-efficiency machining methods for such materials. Observing internal cracks in materials is highly challenging and even requires the assistance of chemical etchants in the observation process. For this reason, numerous scholars have adopted simulation methods to study the machining processes of hard and brittle materials. The finite element method (FEM) is a commonly used simulation technique. Since the Mode I fracture behavior of glass is similar to that of rock, rock fracture models are often adopted for simulating the fracture of glass materials. In FEM simulations, the Rankine criterion is the primary basis for controlling crack initiation, with the crack propagation direction being perpendicular to the maximum principal tensile stress. However, the subsequent propagation and failure behaviors of cracks cannot be fully characterized by the Mode I model; instead, the Mode II model is required to describe these late-stage failure behaviors [15], as shown in Figure 2. The corresponding fracture criteria for Mode I and Mode II cracks are shown in Equations (1) and (2).

(1) $$u_{no}=\frac{2G_{f}^{Ι}}{\sigma_{tu}^{Ι}}$$

where $u_{n0}$ is the critical normal crack-opening displacement at which the tensile stress decreases to zero; $G_{f}^{I}$ is the Mode I fracture energy required to create a unit crack surface; and $\sigma_{tu}^{I}$ is the peak tensile cracking stress in Mode I.

(2) $$\rho (e_{nn}^{ck})={\left(1-\frac{e_{nn}^{ck}}{e_{\max}^{ck}}\right)}^{p}$$

where ρ is the shear-retention factor used to characterize the degradation of shear transfer across the crack; $e_{nn}^{ck}$ is the crack-opening strain; $e_{max}^{ck}$ is the maximum crack-opening strain at which the shear-retention factor approaches zero; *P* is the power-law exponent controlling the degradation rate.

However, the traditional finite element methods rely on finite element meshes, which are prone to mesh distortion when dealing with large deformation problems, resulting in considerable computational cost, and are prone to neglecting and homogenizing microcracks [16,17]. Therefore, conventional FEM may be less suitable for directly capturing discontinuous fracture and microcrack evolution in hard and brittle materials without additional fracture or remeshing techniques. The smoothed particle hydrodynamics (SPH) method does not depend on the mesh, and its particle partitioning method can effectively avoid mesh distortion and accurately respond to crack extension during the processing of hard and brittle materials. Based on this advantage, many researchers have conducted simulation studies on the machining process of hard and brittle materials through the SPH method. Liu et al. [18] simulated the single-particle grinding process by using the SPH method, and researched and analyzed the process of material removal, crack generation and expansion, cutting force and surface roughness, and put forward the three ways of hard and brittle material removal, namely, ductile removal, brittle-assisted removal and brittle removal.

The discrete element method is a computational method that discretizes the computational domain into spherical-shaped particles with contact and bonding models, and its simulation of cracking is based on a large number of contact models between particles [19]. In geotechnical engineering, discrete elements are a common method to study cracking during the fracture of hard and brittle rocks. In order to better study the crack generation and expansion, scholars have used molecular dynamics (MD) and the discrete element method (DEM) to simulate crack evolution [20]. The Finite Element Method (FEM) is the most commonly used simulation tool to study machining processes such as milling, turning and grinding. Zhao et al. [21] developed a finite element model of nanoindentation of 3C-SiC to investigate plastic deformation and brittle fracture during the process and to determine the change in workpiece loading under plastic-ductile transition. Guo et al. [22] investigated the crack extension mechanism of K9 glass under scratch machining based on the SPH method, clarified the relationship between the direction of crack extension and stress, and determined the relationship between the depth of scratch and the depth of crack influence. Meng et al. [23] established a two-dimensional cutting model of 6H-SiC based on molecular dynamics and investigated microscale crack propagation during cutting, the effect of anisotropy on crack distribution, and crack evolution when the machining depth exceeded the ductile–brittle transition threshold. The discrete element method (DEM) is a simulation method that can directly project the actual physical particles and contact states into the particle model under the condition of complete data of system particles and contact information, which can realize the accurate prediction of the mechanical response of the system under various load excitations. Wang et al. [24] simulated the two-dimensional cutting process of rock based on the DEM method and accurately predicted the load changes and crack extension process during the machining process. Jiang et al. [25] simulated the grinding process of SiC ceramic material based on the DEM method, determined the internal contact conditions of the material, and analyzed the relationship between the grinding force and crack extension. Zhang et al. [26] numerically calculated the crack length and the area affected by cracks during vibration-assisted milling machining. Cundall et al. [27] described the discrete element method as a numerical model capable of characterizing the mechanical behavior of assemblies of discs and spheres and elaborated on the key features of this method. Liu et al. [28] established a specific cutting energy model for LTUVAM of borosilicate glass to predict the critical undeformed chip thickness, clarifying the relationship between tool–workpiece contact rate and material ductile–brittle transition.

Existing studies have greatly advanced the understanding of crack propagation and numerical simulation methods for hard and brittle materials. Nevertheless, borosilicate glass machining under the combined action of longitudinal–torsional ultrasonic vibration and adsorption-active media has not been fully investigated. In particular, the relationship among ultrasonic-induced intermittent loading, Rehbinder-effect-assisted surface weakening, crack evolution and machined surface integrity remains unclear. Therefore, further investigation is required to predict crack propagation and clarify surface-quality improvement mechanisms in LTUVAM of borosilicate glass.

In this study, a two-dimensional cutting model of borosilicate glass is established based on the discrete element method, and longitudinal–torsional ultrasonic vibration is incorporated to realize the simulation of crack initiation and propagation processes. LTUVAM experiments combined with the Rehbinder effect are carried out, and the synergistic effects of ultrasonic vibration and surface chemical action are analyzed. By comparing the simulation prediction results with the experimental analysis data, the reliability and applicability of the established model are verified, and the synergistic mechanism of longitudinal–torsional ultrasonic vibration and the Rehbinder effect in crack inhibition and surface quality improvement is elucidated.

## 2. Discrete Element Simulation Method

### 2.1. Principle of the Discrete Element Method

**Figure 3:**
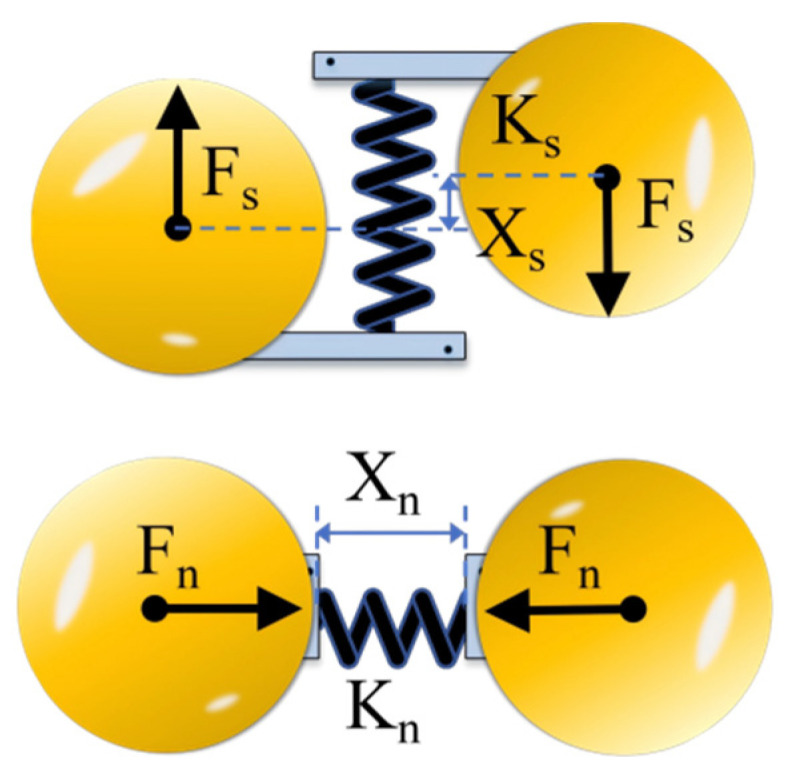
Interparticle bonding model.

The discrete element method (DEM) adopts the soft-ball model, which permits overlap between colliding spheres, and the contact force components in both normal and tangential directions are calculated from the overlap amount [29]. The bonds between spheres in the model rely on breakable springs, and interactive forces are generated when adjacent spheres come into point contact, as illustrated in Figure 3. The dynamic evolution of the model is studied using a time-step iterative algorithm for the motion of discrete particles. The numerical simulation is implemented by applying external forces to selected particles, which cause deformation of the normal and tangential springs between spheres. The normal contact force between bonded particles is expressed in Equation (3). Subsequently, iterative calculations using the time-stepping algorithm are performed to obtain the response, deformation, and damage of the model under specified loading conditions [30].


(3) $$F_{n}=\left\{\begin{array}{l} K_{n}X_{n},(X_{n}<0)\\ K_{n}X_{n},(0\leq X_{n}\leq X_{b})\\ 0,(X_{n}>X_{b}) \end{array}\right.$$

In the formula, $F_{n}$ is the normal force perpendicular to the contact surface, $K_{n}$ the normal stiffness, and $X_{n}$ the relative normal displacement; the value of the normal displacement is the distance between the two spheres minus the sphere diameter. $X_{n}$ denotes the critical normal displacement for fracture. When $X_{n}<0$ the two particles are in a compressed state, with repulsive forces acting between the spheres. When $X_{n}>X_{b}>0$, the normal springs between particles fracture, and the particles are no longer constrained by normal tensile forces [31]. Therefore, the maximum normal force that can be sustained by the normal spring is given by Equation (4).


(4) $$F_{n\max}=K_{n}X_{b}$$

The formula for the critical normal displacement $X_{b}$ for fracture is given in Equation (5).

(5) $$X_{b}=\frac{3K_{n}+K_{s}}{6\sqrt{2}K_{n}(K_{n}+K_{s})}\cdot T_{u}\cdot d^{2}$$

where $T_{u}$ is the tensile strength and $K_{s}$ the shear stiffness.

The normal stiffness $K_{n}$ is calculated using Equation (6).

(6) $$K_{n}=\frac{\sqrt{2}Ed}{4(1-2\nu )}$$

where E is the elastic modulus, d the particle diameter, and ν the Poisson’s ratio. The shear stiffness $K_{s}$ is calculated using Equation (7).


(7) $$K_{s}=\frac{\sqrt{2}(1-5\nu )Ed}{4(1+\nu )(1-2\nu )}$$

For two fully bonded spheres to reach a completely separated state, the calculation of the shear force $F_{s}$ parallel to the contact surface should be considered in addition to the influence of the normal force $F_{n}$ perpendicular to the contact surface, the shear force between the two spheres is represented by a breakable shear spring connection, the formula for the shear spring force $F_{s}$ is given in Equation (8).

(8) $$F_{s}=K_{s}X_{s}$$

where $X_{s}$ is the tangential relative displacement between the two particles.

The maximum shear force $F_{s\max}$ of the shear spring under the Mohr–Coulomb criterion is expressed in Equation (9).

(9) $$F_{s\max}=F_{s0}-\mu_{p}F_{n}$$

where $F_{s0}$ is the maximum shear resistance between particles in the absence of normal force, $\mu_{p}$ is the friction coefficient between particles, and $F_{n}$ is the normal force, with negative values indicating compressive force. The related parameters are further defined in Equations (10) and (11).

(10) $$\mu_{p}=\frac{-2\sqrt{2}+\sqrt{2}l}{2+2l},l={\left[{\left(1+\mu_{i}^{2}\right)}^{1/2}+\mu_{i}\right]}^{2}$$

(11) $$F_{s0}=\frac{1-\sqrt{2}\mu_{p}}{6}\cdot C_{u}\cdot d^{2}$$

where $\mu_{i}$ is the internal friction coefficient and $C_{u}$ the compressive strength.

When the external force is greater than the maximum shear force in the equation, the shear springs between the particles break. At this moment, the shear resistance between the particles no longer exists, and the particles begin to slide relative to each other. The shear force is then expressed in Equation (12).


(12) $$F_{s\max-s}=-\mu_{p}F_{n}$$

The overall mechanical behavior of the particle flow discrete element model mainly depends on the inter-particle contact model and its parameter settings. The above content reflects the correspondence between macroscopic and microscopic mechanics in the model.

### 2.2. Construction of the Discrete Element Model for Borosilicate Glass

**Figure 4:**
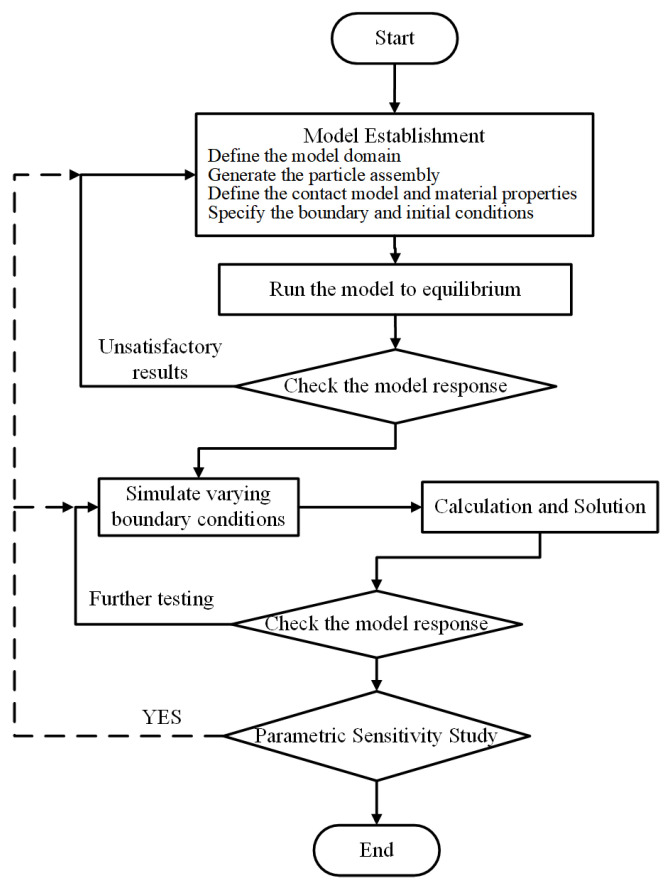
General solution procedure of PFC.

Based on the above principles, numerical simulations of the cutting process of borosilicate glass were carried out using the discrete element software PFC (Particle Flow Code). By accurately describing the contact state between particles in the system, PFC can directly incorporate actual physical particles and their contact conditions into the particle model, thereby accurately predicting the mechanical response of the system under various loading excitations and realizing microscale micromechanical simulations. The PFC simulation mainly consists of four basic components: model domain, particle assembly, contact behavior and material properties, as well as boundary and initial conditions. The simulation flow of PFC is shown in Figure 4.

In this study, BOROFLOAT 33 borosilicate glass was selected as the simulation object. The material properties of borosilicate glass are listed in Table 1. The mechanical parameters of borosilicate glass cannot be directly applied in discrete element numerical simulations. Therefore, calibration of the microparameters for the numerical model is required. In this paper, numerical models for compressive and tensile strength tests were established, and the microparameters were gradually adjusted and calibrated with reference to the experimental conditions [32]. Taking macroscopic mechanical properties such as Young’s modulus, Poisson’s ratio, fracture toughness and Knoop hardness as evaluation criteria, a set of microparameters was obtained through iterative calibration so that the simulated macroscopic responses were consistent with the target mechanical properties of borosilicate glass, as shown in Table 2.

To make the calibration procedure more verifiable, the macroscopic mechanical responses reproduced by the calibrated PFC2D model were further compared with the target properties of BOROFLOAT 33 borosilicate glass. Young’s modulus, Poisson’s ratio and bending strength were selected as the main validation indicators, while fracture toughness was used as a supplementary fracture-related reference for evaluating the brittle-fracture consistency of the calibrated model. The relative error between each model-predicted value and the corresponding target value was calculated to quantitatively evaluate the calibration accuracy. Referring to the commonly used validation practice in PFC/DEM parameter calibration studies, the calibration was considered acceptable when the relative errors of the main macroscopic mechanical parameters were within 5%. As shown in Table 3, the calibrated PFC2D model reproduces the main macroscopic mechanical properties of borosilicate glass with acceptable accuracy, indicating that the micromechanical parameters listed in Table 2 are suitable for the subsequent cutting simulations.

**Figure 5:**
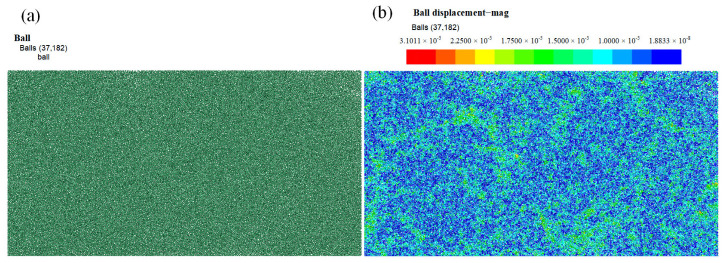
PFC workpiece model. (**a**) particle distribution and (**b**) movable contact condition.

Subsequently, a particle model of the borosilicate glass material was constructed using the PFC2D. The simulation domain was set as a 2 mm × 1 mm two-dimensional plane, with wall constraints applied around the boundaries. The particle radius was set in the range of 2.5 μm to 5.0 μm, and a randomly distributed particle system was generated with a porosity of 0.15. The contact model between particles and walls was defined as the linear stiffness contact model, and the contact behavior between particles was modeled using the Flat-Joint Model, which can effectively simulate typical behaviors such as shear slip, tensile fracture and bond failure. Finally, the particle model of the borosilicate glass material was established, as shown in Figure 5.

## 3. Simulation Results and Analysis

### 3.1. Simulation Results of CM

**Figure 6:**
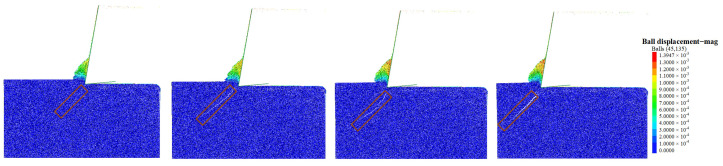
Simulated crack initiation and propagation during CM. Red boxes indicate representative simulated cracks.

The cutting simulation model of borosilicate glass was established using PFC2D based on the discrete element method. With a spindle speed of 24,000 rpm and without external ultrasonic vibration, the crack initiation and propagation process dominated by the brittle failure mode was obtained. As shown in Figure 6, radial splitting cracks are generated at the inter-particle interfaces during the tool advancement along the X-axis, exhibiting a typical crack path propagating forward and downward from the cutting front. Most cracks propagated along the inter-particle boundaries, accompanied by overall spalling and brittle fracture of local particles, indicating insufficient local bonding strength and a material removal mode dominated by brittle fracture. This is consistent with the typical low ductility and high brittleness of borosilicate glass. The results provide a theoretical basis for the subsequent analysis of LTUVAM and LTUVAM under the Rehbinder effect (R-LTUVAM).

### 3.2. Simulation Analysis of LTUVAM

**Figure 7:**
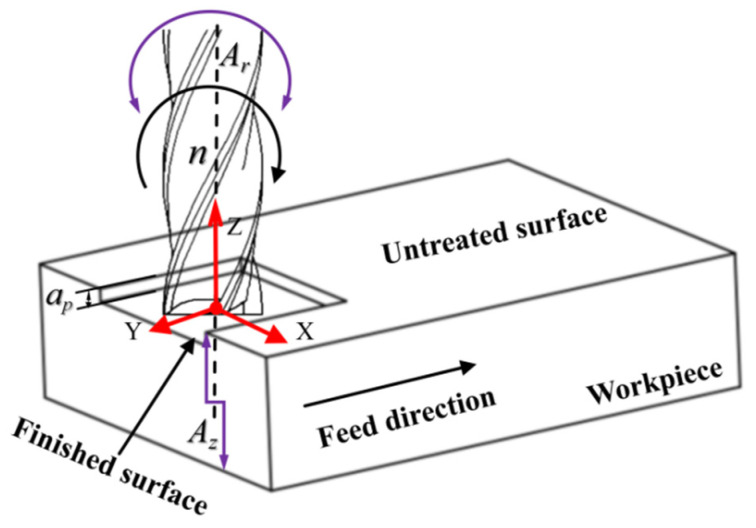
Schematic diagram of LTUVAM.

Based on the established and calibrated DEM model, LTUVAM was simulated, whose process is illustrated in Figure 7 and introduces high-frequency periodic vibrations along the tool axis and tool periphery on the basis of basic tool rotation and feed motion.

In this study, the actual LTUVAM process was simplified as an equivalent two-dimensional tool–workpiece interaction in the PFC2D model. The cutting process was described in the X-Y plane, where the X direction represents the equivalent cutting/feed direction and the Y direction represents the normal/depth direction of the workpiece. The imported cutter wall was treated as a rigid body, and its prescribed motion was imposed through velocity boundary conditions in the PFC program. For clarity, the equivalent displacement components of the tool wall are expressed in Equations (13) and (14), and the corresponding velocity components are given in Equations (15) and (16).

(13) $$x_{T}(t)=x_{T0}+v_{c}t$$

(14) $$y_{T}(t)=y_{T0}+A_{eq}\sin(2\pi f_{us}t)$$

(15) $${\dot{x}}_{T}(t)=v_{c}$$

(16) $${\dot{y}}_{T}(t)=2\pi f_{us}A_{eq}\cos(2\pi f_{us}t)$$

where $x_{T}(t)$ and $y_{T}(t)$ are the coordinates of the tool wall, $v_{c}$ is the equivalent cutting velocity along the X direction, ${\dot{x}}_{T}(t)$ and ${\dot{y}}_{T}(t)$ are the corresponding velocity components imposed on the tool wall in the PFC program, $f_{us}$ is the ultrasonic vibration frequency, and $A_{eq}$ is the equivalent in-plane ultrasonic vibration amplitude. In the present simulation, $f_{us}$ = 26 kHz and $A_{eq}$ = 2 μm. The CM case corresponds to $A_{eq}$ = 0, whereas the LTUVAM case was obtained by superimposing the harmonic perturbation in the Y direction on the translational cutting motion in the X direction.

Because PFC2D is restricted to a two-dimensional plane, the axial longitudinal vibration and the circumferential torsional vibration of the actual rotating tool were not independently resolved. Instead, their combined contact-level effect was represented by an equivalent in-plane harmonic perturbation imposed on the tool wall. Therefore, the amplitude of 2 μm denotes the equivalent in-plane ultrasonic vibration amplitude used in the two-dimensional simulation, rather than an independently measured longitudinal or torsional amplitude. It should also be noted that the present 2D model cannot fully reproduce the actual three-dimensional kinematics of LTUVAM milling, and the simulation results are mainly used to reveal the mechanism-level influence of intermittent ultrasonic loading on crack evolution.

**Figure 8:**
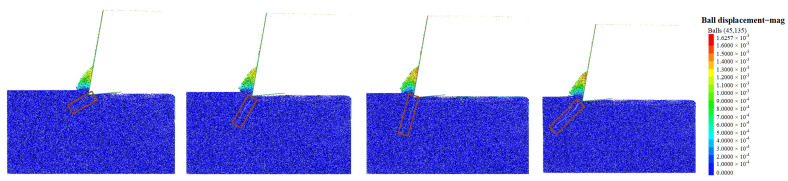
Simulated crack initiation and propagation during LTUVAM. Red boxes indicate representative simulated cracks.

The simulation investigates the disturbance effect of the high-frequency, small-amplitude oscillation superimposed on the main motion on the contact stress state of particles. The simulated crack initiation and propagation characteristics under LTUVAM are shown in Figure 8. To provide a more objective comparison of crack evolution under CM and LTUVAM, the final crack networks shown in Figure 6 and Figure 8 were post-processed using three representative indicators: the number of crack clusters, the maximum crack length, and the maximum damage depth, which characterize crack dispersion, crack-extension severity, and subsurface penetration, respectively. A crack cluster was defined as a spatially connected group of failed contacts, and the maximum damage depth was measured as the vertical distance from the local machined surface to the deepest crack tip. The quantitative results are summarized in Table 4.

As summarized in Table 4, LTUVAM was associated with a larger number of crack clusters but lower maximum crack length and damage depth than CM. This combination suggests that ultrasonic vibration redistributes fracture from localized, deeply penetrating cracks toward shorter and more dispersed microcracks rather than eliminating fracture. The periodic loading–unloading and intermittent tool–workpiece interaction induced by vibration may interrupt sustained crack growth and facilitate localized material separation. This interpretation is consistent with the smaller surface undulations observed in the simulated profile and therefore suggests improved simulated surface integrity under the present cutting conditions.

## 4. Experimental Study on Surface Formation in LTUVAM

### 4.1. Experimental Design of LTUVAM

To investigate how the Rehbinder-active medium further improves the surface quality of borosilicate glass under LTUVAM, comparative milling experiments were designed with surface roughness as the core evaluation index. The experiments focused on comparing LTUVAM without the active medium and R-LTUVAM under the same ultrasonic vibration and cutting parameters. Therefore, the experimental results mainly reveal the additional effect of the Rehbinder-active medium under LTUVAM conditions.

**Figure 9:**
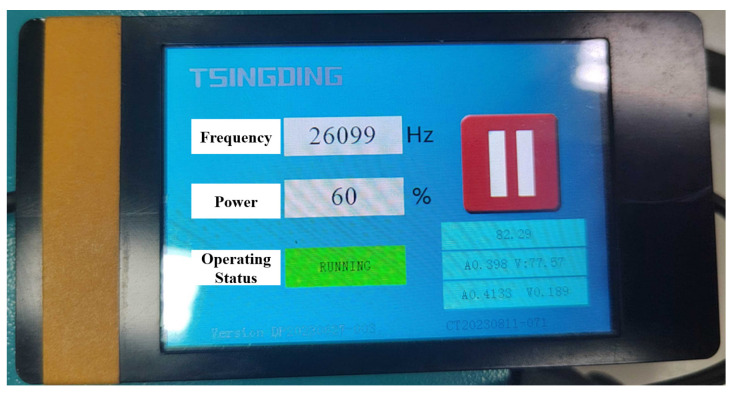
Ultrasonic vibration parameters.

**Figure 10:**
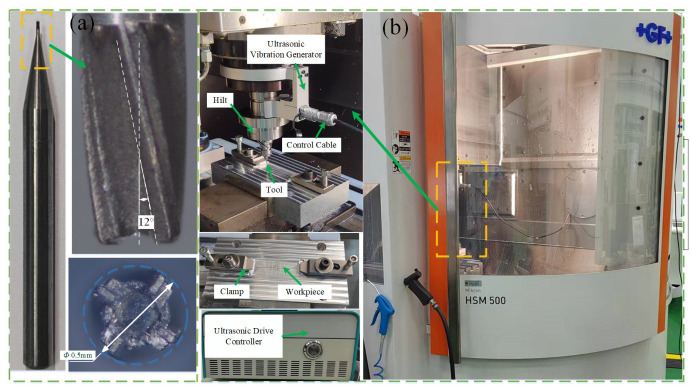
Experimental setup. (**a**) PCD milling cutter; (**b**) experimental machine tool.

**Figure 11:**
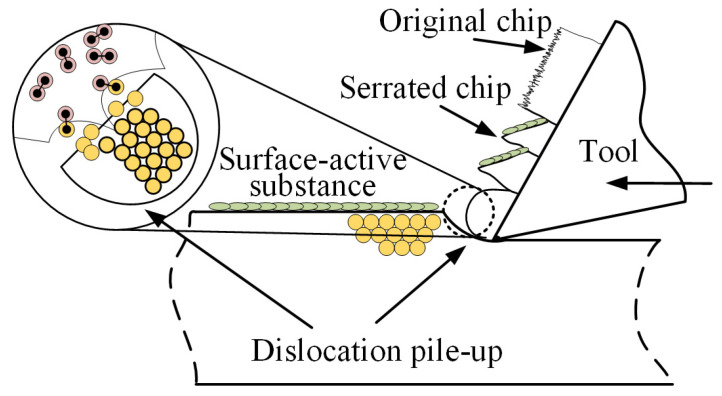
Mechanism diagram of the Rehbinder effect.

In this study, an ultrasonic vibration system driven by a dedicated ultrasonic generator was used, and the ultrasonic frequency was controlled at 26 kHz during machining, as shown in Figure 9. A PCD milling cutter, as shown in Figure 10a, was selected as the cutting tool, and the experimental machine tool is illustrated in Figure 10b. The cutter had a diameter of 0.5 mm, a corner radius of 0.05 mm, and a neck length of 4 mm. A special fixture was used to clamp the workpiece during the experiments. The Rehbinder-active medium used in this study was a water-based glass cutting fluid containing diethanolamine, triethanolamine borate, an organosilicon defoamer, and other functional additives such as wetting agents and bactericides, with deionized water as the solvent. In this study, it was referred to as a Rehbinder-active medium because its amine/borate-containing and surface-active components can improve wetting and adsorption on the borosilicate glass surface and promote a Rehbinder-type surface-active weakening effect during machining. During the experiments, the fluid was supplied to the cutting zone by flushing. Owing to the deep groove in the special fixture, the cutting fluid accumulated around the workpiece and formed a liquid layer covering the machining area. Therefore, no separate pre-immersion treatment was performed before cutting. After each test, visible glass debris and residual fluid were removed from the fixture groove, and the cutting area was cleaned to reduce contamination by glass particles before the next test. It should be noted that the cutting fluid also provided cooling and lubrication, and the term “Rehbinder-active” in this work emphasizes its possible surface-active contribution to crack weakening rather than excluding these auxiliary effects. The mechanism diagram is shown in Figure 11. The ultrasonic vibration power was set at 60% during the milling process, and the feed per tooth was 0.4 μm/z. The spindle speed and milling depth were selected as experimental variables, and the experimental scheme is shown in Table 5. After machining, a Marsurf XT20 3D surface roughness tester was used to measure the 3D surface roughness of the workpiece.

### 4.2. Experimental Results and Analysis

#### 4.2.1. Analysis of 3D Surface Topography

**Figure 12:**
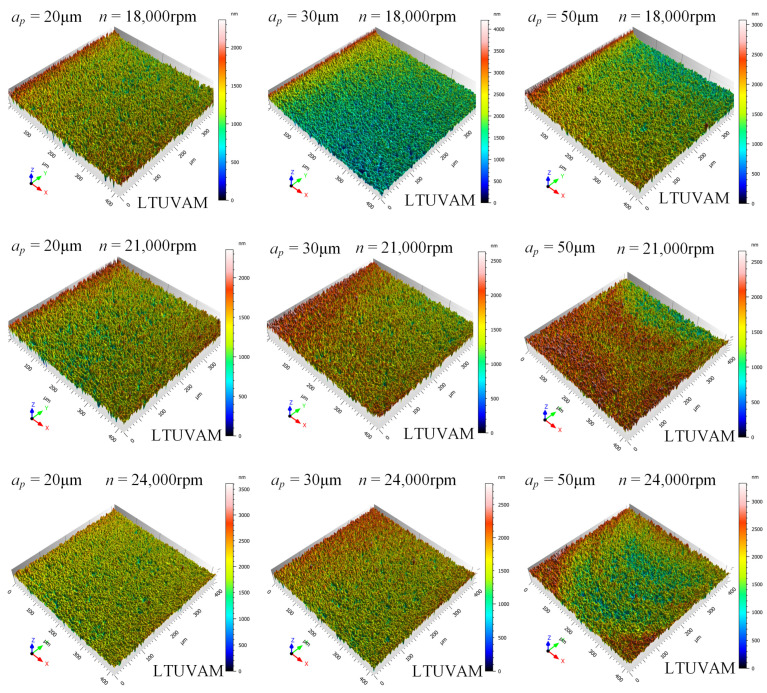
Surface topography obtained by LTUVAM.

**Figure 13:**
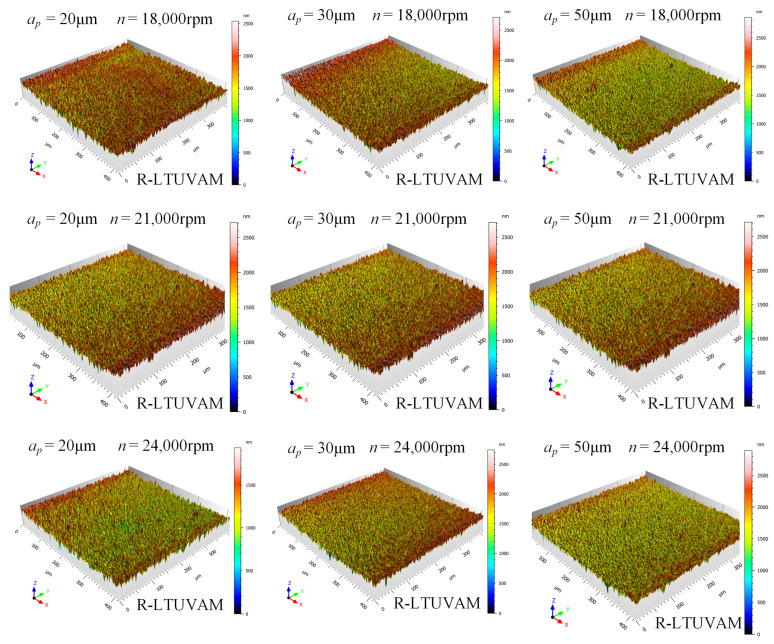
Surface topography obtained by Rehbinder-effect-assisted LTUVAM.

**Figure 14:**
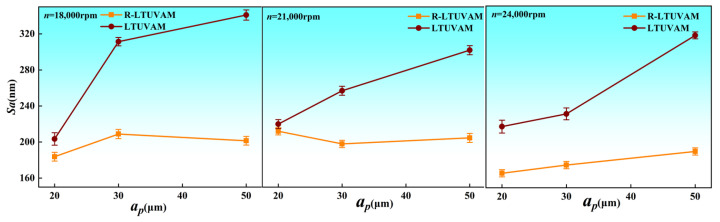
Measured surface roughness under different spindle speeds and axial depths of cut.

The milling surface topographies before and after the introduction of the Rehbinder-active medium are compared in Figure 12 and Figure 13, and the experimental results are summarized in Figure 14. Under the same spindle speed and process parameters, R-LTUVAM produced smaller surface micro-undulations and smoother contours, resulting in an overall improvement in surface quality. This improvement is mainly attributed to the introduction of specific surface-active substances into the cutting zone by the Rehbinder effect during machining. These surface-active substances can reduce the surface energy and locally weaken the bonding strength and fracture resistance of the workpiece material, thereby facilitating material removal and reducing crack-related surface defects as inferred from the smoother surface morphology. In addition, the lubrication and auxiliary cooling effects of the surfactant also help to reduce friction and thermal effects between the tool and workpiece, further minimizing machining-induced surface defects and microcracks.

#### 4.2.2. Analysis of 2D Surface Topography

To further analyze the influence of cutting depth on surface quality, the experimental group at *n* = 24,000 rpm was selected for detailed comparison because this speed produced the most pronounced roughness reduction in Figure 14. The experimental results at this speed exhibited a relatively stable trend in surface quality variation under different cutting depths, which helps clarify the effect of cutting depth on surface formation. Moreover, for the ultrasonic-assisted machining of hard and brittle materials, a high spindle speed is beneficial for reducing the unit cutting thickness and instantaneous cutting force, inhibiting the propagation of material microcracks, and improving machining stability and trajectory consistency [34], thereby better revealing the improvement effect of the Rehbinder effect on surface quality.

**Figure 15:**
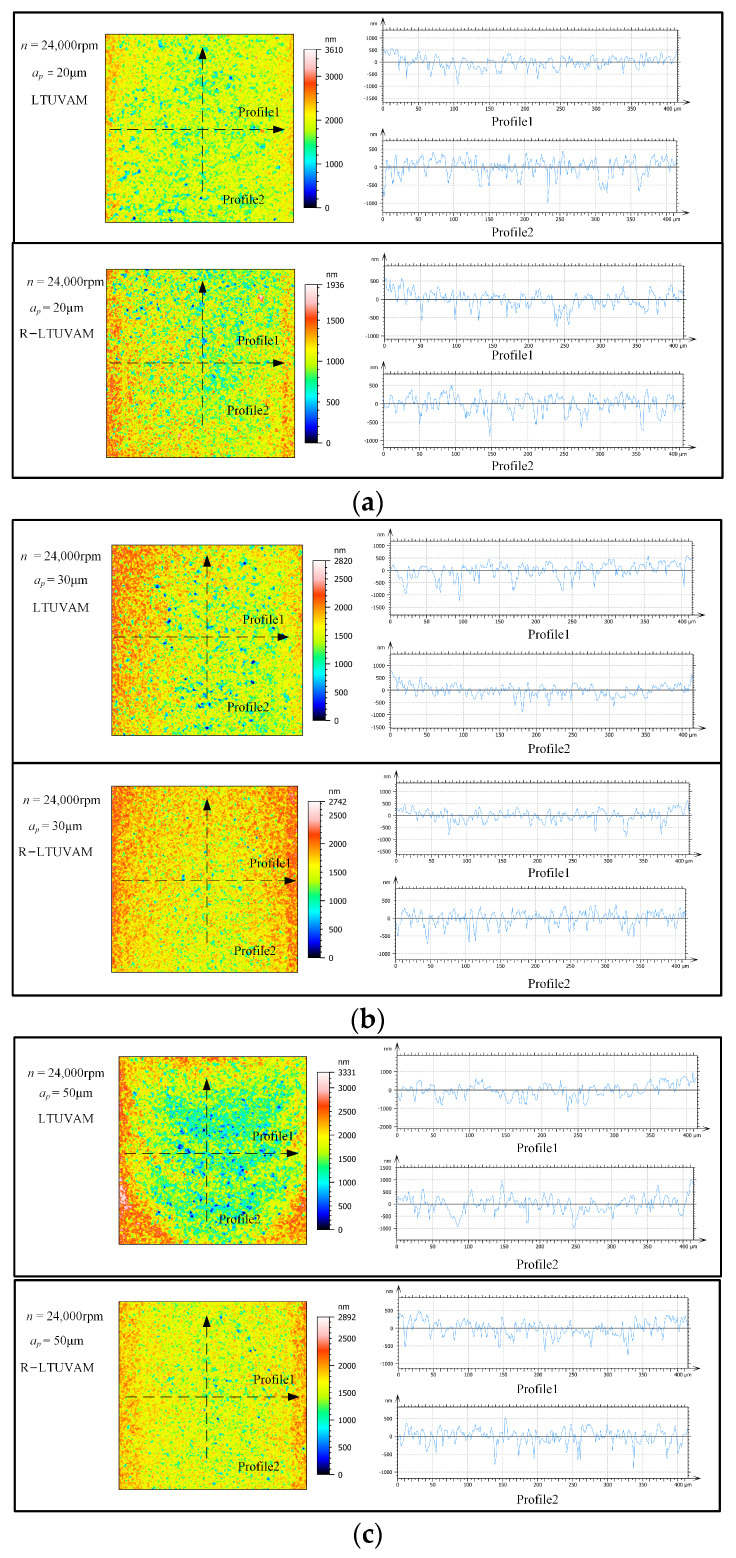
2D surface topography of glass by R-LTUVAM and LTUVAM. (**a**) *n* = 24,000 rpm, *a_p_* = 20 μm; (**b**) *n* = 24,000 rpm, *a_p_* = 30 μm; (**c**) *n* = 24,000 rpm, *a_p_* = 50 μm.

As the cutting depth increased, the surface roughness of the machined surfaces increased for both LTUVAM and R-LTUVAM, indicating gradual deterioration in surface quality, as shown in Figure 15. This is mainly because a larger depth of cut increases the instantaneous material removal volume and cutting load, thereby intensifying brittle fracture, crack propagation, and surface defects. However, under different cutting depth conditions, the fluctuation degree of the workpiece surface obtained by R-LTUVAM is significantly smaller than that of LTUVAM; the curves in both directions are overall smoother, with a relatively small range of contour variation, reflecting a lower residual height distribution. In particular, as the cutting depth gradually increases, as shown in Figure 15c (when the cutting depth is 50 μm), the contour curve of LTUVAM exhibits relatively sharp peaks and deep valleys, showing a large range and strong surface discontinuity; in contrast, the fluctuation amplitude of the contour curve under R-LTUVAM is relatively weakened, without abrupt profile fluctuations, and the overall surface micro-morphology tends to be more uniform. This indicates that, with the assistance of the Rehbinder effect, both the maximum peak height and the minimum valley depth of the material surface are reduced. This result further demonstrates the potential of the Rehbinder effect for improving the surface quality and machining stability of ultrasonic-assisted milling.

### 4.3. Analysis of Surface Roughness

**Figure 16:**
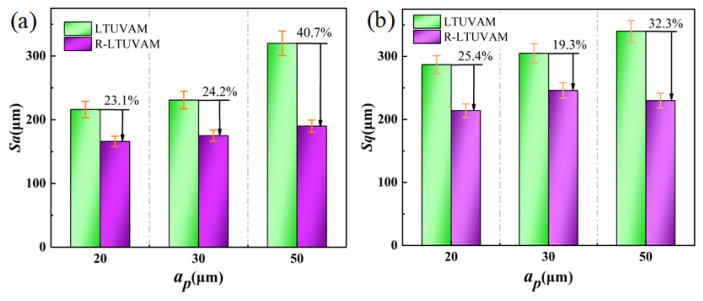
Effect of axial depth of cut on surface roughness at *n* = 24,000 rpm. (**a**) arithmetic mean height *S_a_*; (**b**) root mean square height *S_q_* (the error bars represent the 95% confidence intervals).

To further illustrate the detailed trend of R-LTUVAM on the surface of borosilicate glass, the case with a spindle speed of 24,000 rpm was selected for analysis. As shown in Figure 14, the surface roughness value decreased most significantly at this speed, indicating that the introduction of the Rehbinder-active medium provided an additional improvement in surface quality under the LTUVAM condition. The experimental data of the surface roughness parameters for LTUVAM and R-LTUVAM at this spindle speed are presented in Figure 16. As shown in Figure 16, under the same spindle speed, the surface roughness of the workpiece increased with increasing depth of cut. Under the same experimental conditions, the surface roughness of the workpiece machined by R-LTUVAM was significantly lower than that by LTUVAM. In particular, when the cutting depth was 50 μm, the *S_a_* value decreased by 40.7% and the *S_q_* value decreased by 32.3%. The trend analysis of roughness at different cutting depths in the range of 18,000–24,000 rpm is shown in Figure 14. It can be concluded that under the same cutting depth condition, the higher the spindle speed, the lower the surface roughness. This indicates that appropriately increasing the spindle speed helps achieve more stable material removal, effectively inhibits microcrack propagation and surface-defect formation, and thereby further improves the machined surface quality.

### 4.4. Qualitative Assessment of the Milling Simulation Model

**Figure 17:**
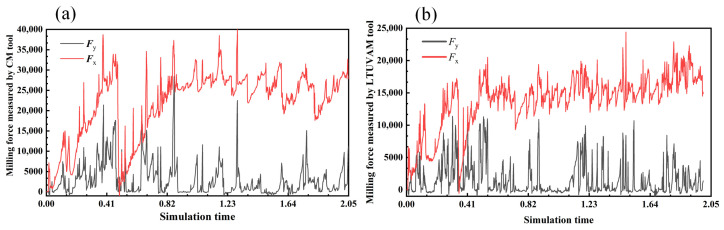
Simulated milling-force histories. (**a**) CM; (**b**) LTUVAM.

The milling force curves in the simulation results are shown in Figure 17. Under CM conditions, the contact process between the tool and workpiece was continuous and stable, and the milling-force curve was generally smooth with a high amplitude. This indicates that material removal was dominated by brittle fracture during cutting and that the tool bore a relatively large load. After introducing the longitudinal–torsional ultrasonic vibration, both the normal and tangential force curves of the tool obtained from the simulation exhibited obvious periodic fluctuation characteristics, with a significant reduction in force peaks and the appearance of regular instantaneous unloading intervals. This mechanical characteristic of “periodic loading-rapid unloading” indicates that an intermittent cutting action is formed between the tool and the material, which effectively weakens the stress concentration and promotes material removal in the form of brittle spalling.

Furthermore, the applicability of the PFC2D model was qualitatively assessed by comparing the simulated mechanical response and crack-evolution tendency with reported findings and the experimental surface-formation results. The simulation results show that the introduction of longitudinal–torsional ultrasonic vibration reduces the peak milling force and produces periodic loading–unloading characteristics during tool–workpiece interaction. This intermittent cutting behavior weakens stress concentration at the tool front and promotes the formation of finer and more uniformly distributed cracks, thereby reducing the severity of localized crack extension. This simulated tendency is consistent with previous studies showing that ultrasonic vibration can reduce cutting force and improve machined surface quality [35,36]. From the experimental perspective, the comparison between LTUVAM and R-LTUVAM further showed that the introduction of the Rehbinder-active medium led to lower roughness values and smoother surface profiles under the same LTUVAM conditions. This surface-quality improvement is consistent with the mechanism-level tendency revealed by the simulation, namely that a more stable tool–workpiece interaction and reduced localized crack-extension severity are beneficial to surface formation. Therefore, the PFC2D model can reasonably reflect the mechanism-level mechanical response and crack-evolution characteristics of borosilicate glass during LTUVAM. The model can be used as a theoretical tool for further investigating the LTUVAM mechanism and for preliminary optimization of machining parameters for hard and brittle materials.

## 5. Conclusions

This paper investigated the problem of crack generation and surface quality deterioration during milling of borosilicate glass. A two-dimensional cutting model was established based on the discrete element method, and the initiation and propagation behavior of cracks were systematically analyzed. Combined with R-LTUVAM experiments, the influence of process parameters on surface quality was investigated. The applicability of the model is qualitatively supported by the consistency between the simulation results, experimental roughness trends, and reported findings. This work provides a theoretical basis and process guidance for the efficient and precise machining of hard and brittle materials. The conclusions are as follows:

(1) A two-dimensional DEM of borosilicate glass was established based on PFC2D to simulate crack initiation and propagation in CM and LTUVAM. The results show that radial splitting cracks and particle spalling occurred during material brittle fracture, and the simulated cutting force was reduced under LTUVAM.

(2) LTUVAM experiments were conducted to investigate the additional effect of the Rehbinder-active medium and process parameters on surface roughness under LTUVAM conditions. With the Rehbinder-active medium, surface roughness was significantly reduced, especially at a cutting depth of 50 μm, where *S_a_* and *S_q_* decreased by 40.7% and 32.3%, respectively. This result indicates improved surface formation and indirectly suggests a reduction in crack-related surface damage under R-LTUVAM conditions.

(3) The simulated cutting-force variation and crack-evolution tendency were qualitatively compared with the experimental surface-roughness trends and reported results. The consistency in mechanism-level trends supported the validity of the PFC2D model for revealing crack-evolution and material-removal tendencies during LTUVAM of borosilicate glass. The model can reflect the mechanical response and crack evolution under ultrasonic vibration at the mechanism level and can be further used for mechanism research and preliminary parameter optimization of LTUVAM of glass materials.

## Figures and Tables

**Table 1:** Properties of borosilicate glass materials.

Properties	Value
Chemical composition	SiO_2_, B_2_O_3_, Na_2_O/K_2_O, Al_2_O_3_
Density	2.23 g/cm^3^
Young’s modulus	64 GPa
Poisson’s ratio	0.2
Knoop hardness	480
Bending strength	25 MPa
Fracture toughness	0.7 MPa∙m^1/2^ [33]

**Table 2:** Micromechanical parameters used in the PFC2D model.

Properties	Value
Normal stiffness	8.1 × 10^10^ Pa/m
Shear stiffness	2.025 × 10^10^ Pa/m
Friction coefficient	0.577
Normal bond strength	1.69 × 10^8^ Pa
Shear bond strength	1.85 × 10^8^ Pa
Damping coefficient	0.7

**Table 3:** Validation of the calibrated PFC2D model for borosilicate glass.

Mechanical Property	Target Value	Model Value	Relative Error (%)
Young’s modulus (GPa)	64.0	63.1	1.41
Poisson’s ratio	0.20	0.195	2.50
Bending strength (MPa)	25.0	24.2	3.20
Fracture toughness (MPa∙m^1/2^)	0.70	0.68	2.86

**Table 4:** Quantitative comparison of simulated crack characteristics: CM vs. LTUVAM.

Machining Condition	Total Number of Crack Clusters	Maximum Crack Length (μm)	Maximum Damage Depth (μm)
CM	43	186.5	82.7
LTUVAM	71	98.2	39.6

**Table 5:** Experimental parameters for glass milling.

*n*	*a_p_*	Rehbinder Effect	Ultrasonic Power	*f_z_*
rpm	μm		%	μm/z
18,000	20	Present	60	0.4
		Absent		
	30	Present		
		Absent		
	50	Present		
		Absent		
21,000	20	Present	60	0.4
		Absent		
	30	Present		
		Absent		
	50	Present		
		Absent		
24,000	20	Present	60	0.4
		Absent		
	30	Present		
		Absent		
	50	Present		
		Absent		

## Data Availability

The original contributions presented in this study are included in the article. Further inquiries can be directed to the corresponding author.
